# Preferable outcome of Janus kinase inhibitors for a group of difficult-to-treat rheumatoid arthritis patients: from the FIRST Registry

**DOI:** 10.1186/s13075-022-02744-7

**Published:** 2022-03-01

**Authors:** Sae Ochi, Koshiro Sonomoto, Shingo Nakayamada, Yoshiya Tanaka

**Affiliations:** 1 Department of Laboratory Medicine, The Jikei University School of Medicine, Nishi-shinbashi 3-25-8, Minatoku, Tokyo, 105-8461 Japan; 2 The First Department of Internal Medicine, School of Medicine University of Occupational and Environmental Health, Japan, Iseigaoka1-1, Yahata-Nishiku, Kitakyushu, 807-8555 Japan

**Keywords:** Difficult-to-treat rheumatoid arthritis, Biologic and targeted synthetic disease-modifying anti-rheumatic drugs, Janus kinase inhibitor

## Abstract

**Backgrounds:**

Treatment of difficult-to-treat rheumatoid arthritis (D2T RA) is one of the greatest unmet needs in rheumatology. This study aims to find out preferable treatment options for a group of D2T RA patients who are refractory to multiple biologic and targeted synthetic disease-modifying anti-rheumatic drugs (b/tsDMARDs).

**Methods:**

Data were obtained from patients enrolled in the FIRST Registry who started either TNF inhibitor (TNFi), interleukin-6 receptor inhibitor, cytotoxic T-lymphocyte–associated antigen-4 immunoglobulin, or Janus-kinase inhibitor (JAKi) in the period of August 2013 to December 2020. Those who failed to ≥ 2 and ≥ 3 b/tsDMARDs were categorised as D2T RA and very D2T RA (vD2T RA), respectively. Change in Clinical Disease Activity Index (CDAI) and Health Assessment Questionnaire Disability Index were compared among the groups using propensity-based inverse probability treatment weighted (IPTW) method.

**Results:**

Of 2128 cases included, 353 were categorised as D2T RA. Among the D2T RA, 106 were identified as vD2T RA. JAKi showed a significant improvement in CDAI in the patients with D2T RA and vD2T RA, compared to IPTW-adjusted patients treated with the other 3 regimens. Latent class analysis of the trajectories of treatment response revealed that the proportion of a group of patients who showed poor response was lower among the JAKi subgroup than among those with other subgroups. This superiority of JAKi was more apparent among methotrexate- and glucocorticoid-free individuals. The hazard ratio of severe adverse events was comparable among the four treatment subgroups in both the D2T RA and b/tsDMARD-naïve groups.

**Conclusions:**

This study compared responsiveness to different classes of b/tsDMARDs among D2T RA and vD2T RA patients who were refractory to multiple b/tsDMARDs. The results suggest JAKi is a preferable treatment choice for this type of D2T RA.

**Supplementary Information:**

The online version contains supplementary material available at 10.1186/s13075-022-02744-7.

## Background

The recent development of biologic and targeted synthetic disease-modifying anti-rheumatic drugs (b/tsDMARDs) has revolutionised the treatment of rheumatoid arthritis (RA). Evidence from around the world indicate that 30 to 60% of RA patients refractory to their first DMARD can achieve clinical remission following treatment with additional bDMARDs, and structural remission can be achieved in approximately 60 to 90% of patients treated with tumour necrosis factor inhibitors (TNFis) and methotrexate (MTX) [1]. Nonetheless, a proportion of patients are refractory to multiple treatments despite increasing treatment options. This type of RA, called difficult-to-treat RA (D2T RA), is considered as one of the greatest unmet needs in the field of rheumatology [2].

The European Alliance of Associations for Rheumatology (EULAR) launched a new definition of D2T RA in 2020 [3] and points to consider for its management in 2022 [4]. The definition comprises 3 criteria: treatment failure history, characterisation of active/symptomatic disease, and clinical perception. This definition provides us a holistic view of D2T RA patients, and future research may need to use this definition to collect D2T RA patients. In addition, as each of these 3 criteria may reflect different pathologies, epidemiological research may need to be conducted separately for each criterion or may include each criterion as an independent explanatory factor. In the criteria, a major clinical challenge is the treatment of patients who fulfilled the condition of the first criteria: patients who are refractory to multiple b/tsDMARDs with different mechanisms of action. Rheumatologists should particularly want to know the preferable treatment choice for this type of D2T RA patients. To date, several studies have shown preferable outcomes of agents for D2T RA [5]. However, to our knowledge, there is no cohort study directly comparing the effectiveness of different drugs. Therefore, this research aimed to determine which class of b/tsDMARD is more effective and safer for patients who were refractory to multiple b/tsDMARDs. Our findings will provide insight into the best management strategy for D2T RA patients, which is a major goal for many rheumatologists.

## Methods

### Data source

The FIRST Registry is a multi-institutional cohort of RA patients treated with b/tsDMARDs, established by the University of Occupational and Environmental Health, Japan, and its multiple affiliated hospitals. Detail of the cohort is available in other articles [6–10]. In this registry, all registered RA patients were enrolled in a long-term observational study at the point of new or switch-prescription of b/tsDMARDs. If a patient was treated with several b/tsDMARDs, each episode was treated as an independent episode.

By December 2020, 4115 cases were enrolled in the registry. b/tsDMARDs with the following four different mechanisms of action (classes) were included:

- TNFis: infliximab, etanercept, adalimumab, golimumab, and certolizumab
- Interleukin-6 receptor inhibitors (IL-6Ris): tocilizumab and sarilumab
- Cytotoxic T-lymphocyte–associated antigen-4 immunoglobulin (CTLA4-Ig): abatacept
- Janus kinase inhibitors (JAKis): tofacitinib, baricitinib, peficitinib, and upadacitinib.
- Biosimilar bDMARDs were also included. Rituximab was not included in this study because this drug is not approved as a treatment option for RA in Japan.

At the start of b/tsDMARD treatment, baseline data were collected, including demographics, disease duration, titres of anti-cyclic citrullinated protein antibody (ACPA), measures of disease activity, functional status, present and past treatments, serum creatinine levels, and coexistence of chronic lung diseases including chronic bronchitis, bronchial ectasia, interstitial pneumonia, old tuberculosis, and inflammatory lung nodule, and the names of coexisting diseases such as osteoporosis with fracture and chronic heart failure. Follow-up data on disease activity were collected at 2 weeks, 6 months, and 1 year after the start of the therapy. If treatment was discontinued within a year due to severe adverse events (SAEs), data about the date and the reason for treatment cessation were also collected.

### Patient selection and data collection

#### Eligibility criteria

As outcomes of the treatment may differ when treatment options are limited, this study included only cases that were enrolled in the FIRST Registry after JAKis were first approved in Japan—from August 2013 to December 2020.

#### Exclusion criteria

To remove cases who administrated b/tsDMARDs as a part of the treatment of comorbidities (e.g. interstitial lung diseases or vasculitis), cases having both significant comorbidities and prednisolone > 15 mg/day were excluded from the analysis. Information about the coexisting diseases was collected.

### Definition of D2T RA, very D2T RA, and SAE

Based on the EULAR definition, cases that failed to achieve the treatment target with ≥ 2 classes of b/tsDMARDs were identified as D2T RA. In addition to this definition, we categorised cases that failed ≥ 3 classes of b/tsDMARDs as very D2T RA (vD2T RA). Cases treated with a b/tsDMARD for the first time were assigned to the b/tsDMARD-naïve group.

Adverse reactions (e.g. allergic reactions, infections, malignancies, lymphoproliferative disorders, major adverse cardiovascular events, and abnormality in laboratory tests) which lead to treatment discontinuation were recorded as SAEs.

### Statistical analysis

#### Simple comparison of patient background

Backgrounds of the patients in each treatment subgroup were compared across the 4 classes of b/tsDMARDs using one-way analysis of variance (ANOVA) for numerical variables and chi-square test for categorical variables.

#### Latent class analysis

To identify different patterns of drug response, latent class analysis was conducted using the gsem suite of functions in Stata 16 (StataCorp, College Station, TX), which categorised patients into classes. We estimated 3 classes according to previous studies [7, 9]. The number and percentage of cases that fell in a particular latent class were calculated for each treatment type.

#### Panel analysis

Change in CDAI and Health Assessment Questionnaire Disability Index (HAQ-DI) over the course of a year was compared using longitudinal panel data analysis. Regression analyses were conducted using the xt suite of functions in Stata16. A mixed-effects regression model was fitted with age, gender, disease duration, CDAI at week 0, positivity of ACPA, coexistence of pulmonary diseases, and serum creatinine as fixed effects and use of MTX and glucocorticoids as random effects.

### Analyses using a propensity-based inverse-probability treatment weighted method

As the number of patients included for each group (D2T RA and vD2T RA groups) was limited, multivariate regression analysis tended to overfit the data, resulting in a biassed estimation. Therefore, propensity-based inverse probability treatment weighted (IPTW) method was also performed for sensitivity analysis. For CDAI and HAQ-DI, delta (*D*) CDAI and DHAQ-DI were calculated, respectively, as follows and then used for outcomes.

$$\Delta \mathrm{Value}=\left(\mathrm{Value}\ \mathrm{at}\ 1\mathrm{year}\right)-\left(\mathrm{Value}\ \mathrm{at}\ \mathrm{day}\ 0\right)$$

A regression model was used to adjust for potential confounders. Variables that showed a significant correlation with failures to ≥ 2 classes of b/tsDMARDs were included as covariates. Missing data including loss to follow-up were managed as blank, and no imputation was conducted because of homogeneity of the data.

### Analysis of hazards of SAE

Development of SAEs is another major factor that causes D2T RA status. Therefore, Cox regression analysis controlling for age, gender, dose of MTX, and glucocorticoid at day 0 was conducted to assess the hazards of SAE over the course of a year among each treatment subgroup. As the risk of SAEs may differ depending on past failures of b/tsDMARDs, the risk among the b/tsDMARD-naïve group was also analysed. Nelson-Aalen cumulative hazard model was also used to show the time trend of hazard accumulation visually.

## Results

**Fig. 1:**
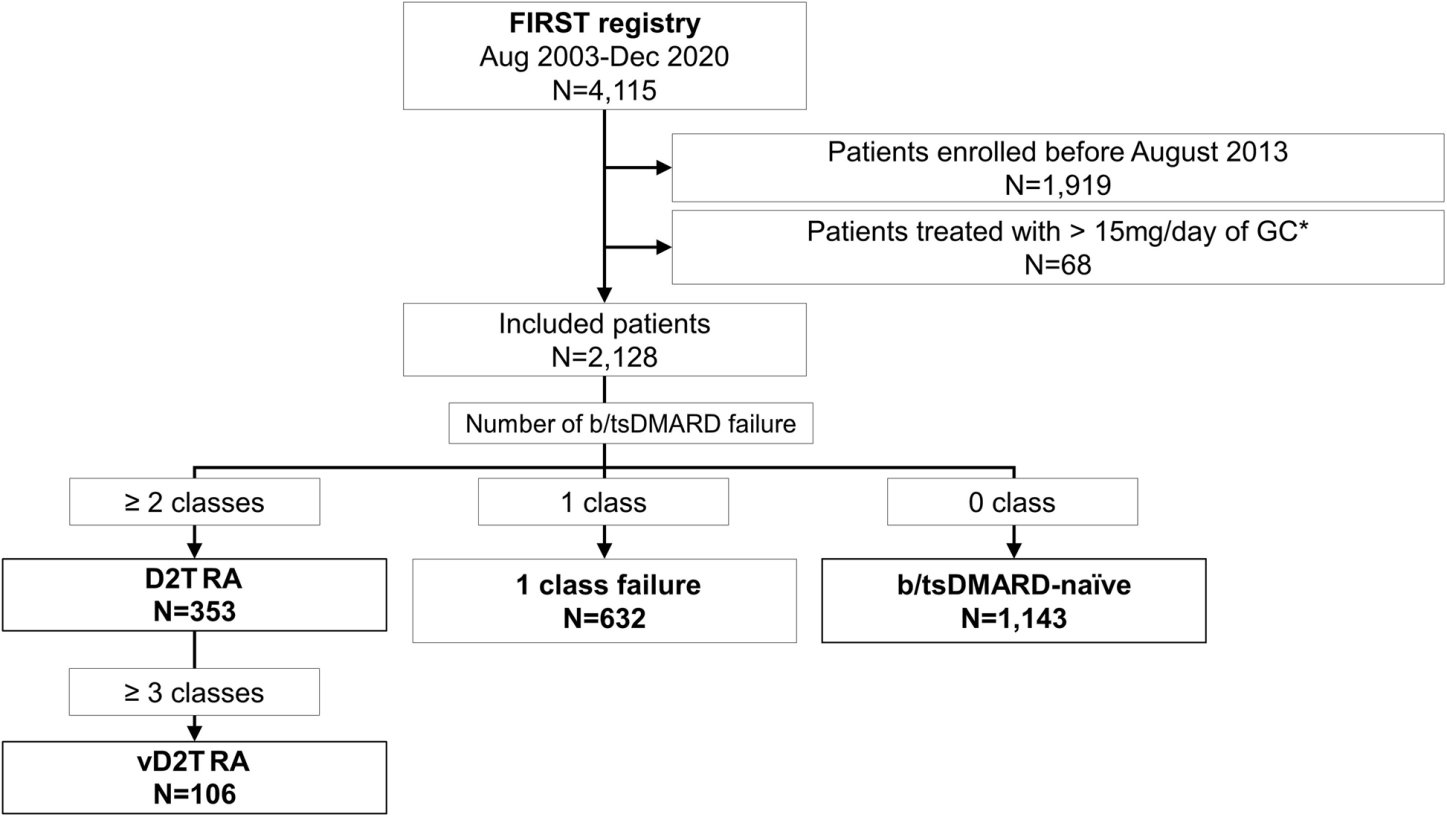
Patient selection. *Prednisolone equivalent dose. GC, glucocorticoid; D2T RA, difficult-to-treat rheumatoid arthritis; vD2T RA, very difficult-to-treat RA; b/tsDMARD, biologic and targeted synthetic disease-modifying anti-rheumatic drugs

By December 2020, 4115 cases were enrolled in the FIRST Registry, of which 1911 cases were enrolled before August 2013 and thus were excluded from this study. Another 68 were treated with a > 15 mg/day prednisolone equivalent dose of glucocorticoids. The breakdown of the reasons of high dose of glucocorticoids is listed in Additional file 1. As a result, 2128 cases were included for further analyses, among which 353 cases had histories of failure for ≥ 2 classes of b/tsDMARDs and were categorised into the D2T RA group. Six hundred and thirty-two and 1143 cases were categorised as the one class failure and the b/tsDMARD-naïve groups, respectively. Among the D2T RA cases, 106 were vD2T RA (Fig. 1).

**Fig. 2:**
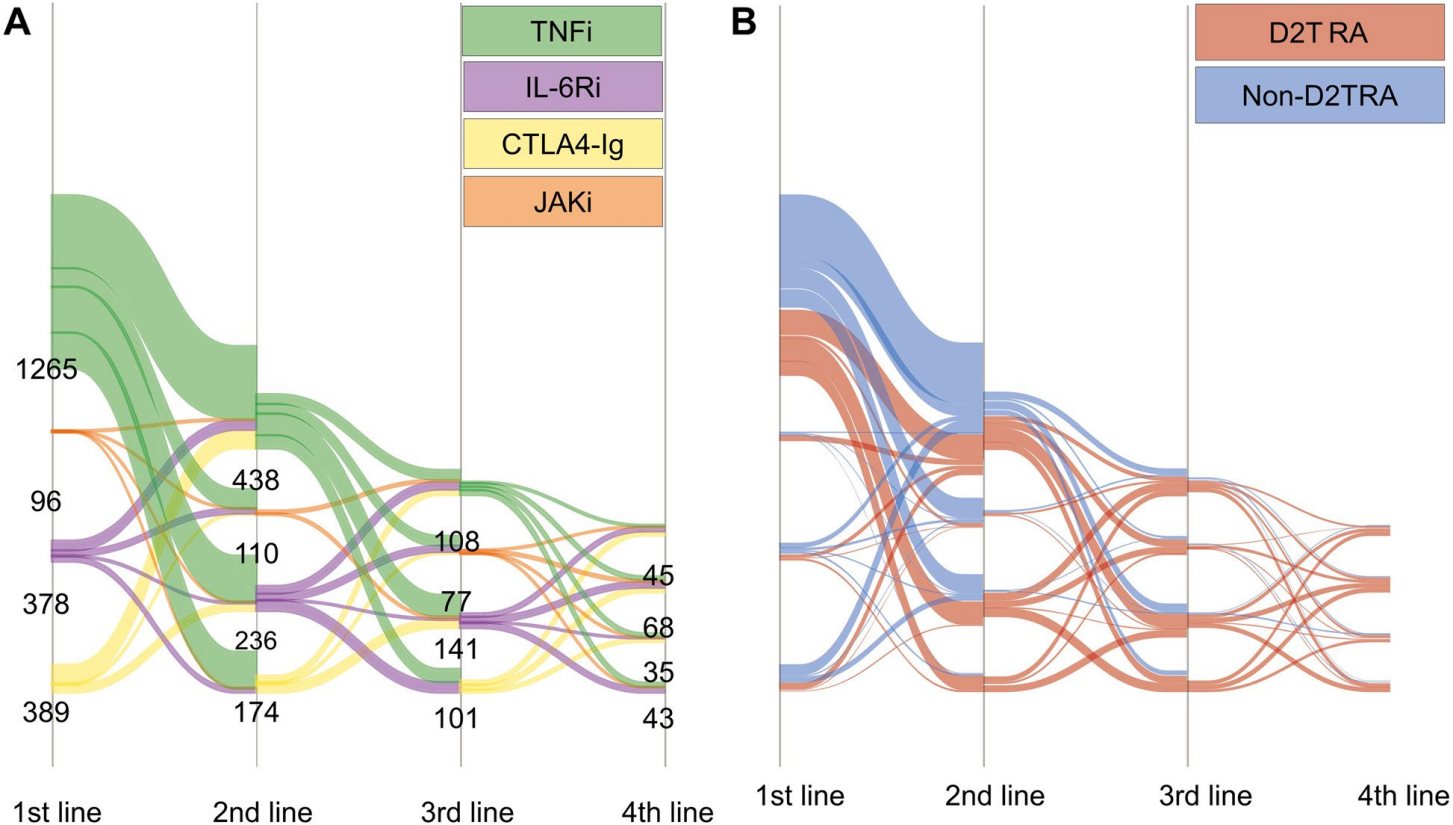
Patterns of the treatment switches in D2T RA cases. Sankey diagram of the patients who participated in the FIRST Registry from August 2013 to January 2021 (*N* = 2176) **A** coloured by treatment type and **B** coloured by D2T RA. D2T RA, difficult-to-treat rheumatoid arthritis; TNFi, tumour necrosis factor inhibitor; IL-6Ri, interleukin-6 receptor inhibitor; CTLA4-Ig, cytotoxic T-lymphocyte-associated antigen-4 immunoglobulin; JAKi, Janus kinase inhibitor

Treatment patterns of these cases are shown in Fig. 2. TNFi was the most commonly used as the first line treatment of b/tsDMARD (Fig. 2A), but for the third and the fourth treatment, all four classes of b/tsDMARDs were used equally in both D2T RA and non-D2T RA cases (Fig. 2B and Additional file 2).

### Patient background

Among the D2T RA group, 71, 79, 58, and 145 cases were included in the TNFi, IL-6i, CTLA4-Ig, and JAKi subgroups, respectively. Demographic and medical backgrounds of the included cases are shown in Table 1. The average age was higher among those receiving IL-6Ri and CTLA4-Ig than those receiving JAKi. There was no significant difference in CDAI among these treatment subgroups. The average serum creatinine level was higher among the TNFi subgroups than among the IL-6Ri and JAKi subgroup. There was no significant difference in the coexistence of chronic lung diseases. The proportion of vD2T RA was higher among the JAKi and TNFi subgroups than among other subgroups, suggesting that these drugs were more likely to be chosen as treatment options for vDt2RA.

**Table 1:** Comparison of the numerical background of D2T RA cases by treatment types. Subgroups are compared using one-way analysis of variance (ANOVA) for numerical variables and chi-squared test for categorical variables

		TNFi (***N*** = 71)			IL-6Ri (***N*** = 79)			CTLA4-Ig (***N*** = 58)			JAKi (***N*** = 145)			***p***
		Mean	SE	Median	Mean	SE	Median	Mean	SE	Median	Mean	SE	Median	
**Numerical variables**														
**Age**		63.5	1.8	67	68.6	1.2	70	64.6	1.6	67.5	60.5	1.1	63	< 0.01*
**Duration (months)**		163.3	13.3	155	169.8	14.0	136	182.9	16.8	143	164.3	9.6	123	0.76
**CDAI**		27.4	1.5	26.2	28.9	1.6	26.2	26.7	1.4	24.8	26.2	1.0	25	0.5
**HAQ-DI**		1.6	0.1	1.5	1.6	0.1	1.6	1.6	0.1	1.6	1.4	0.1	1.4	0.19
**DAS28-CRP**		4.9	0.1	5.0	5.0	0.1	4.7	4.6	0.1	4.5	4.5	0.1	4.6	0.05
**DAS28-ESR**		5.7	0.1	5.8	5.9	0.1	5.8	5.3	0.2	5.2	5.2	0.1	5.4	< 0.01*
**GC dose (mg/day)**^**a**^		1.2	0.3	0	1.3	0.3	0	1.5	0.3	0	1.1	0.2	0	0.75
**MTX dose (mg/week)**		5.5	0.7	6	4.9	0.6	0	6.0	0.9	5	7.1	0.5	8	0.04
**Creatinine (mg/dL)**		0.9	0.1	0.68	0.9	0.1	0.76	0.7	0.0	0.64	0.7	0.0	0.65	0.03*
**Categorical variables**		***N***		**%**	***N***		**%**	***N***		**%**	***N***		**%**	***p***
**Gender**	**Male**	9		12.7	12		15.2	7		12.1	24		16.6	0.81
	**Female**	62		87.3	67		84.8	51		87.9	121		83.4	
**ACPA**	**Negative**	10		14.1	17		21.5	9		15.5	34		23.4	0.36
	**Positive**	57		80.3	57		72.2	49		84.5	111		76.6	
**Disease activity**	**LDA**	1		1.4	4		5.1	0		0.0	10		6.9	0.53
	**MDA**	26		36.6	27		34.2	21		36.2	49		33.8	
	**HDA**	44		62.0	48		60.8	37		63.8	85		58.6	
**Use of MTX**		38		53.5	39		49.4	31		53.4	92		63.4	0.18
**Use of GC**		19		26.8	16		20.3	19		32.8	29		20.0	0.20
**Chronic lung disease**		25		35.2	32		40.5	20		34.5	41		28.3	0.31
**Chronic heart disease**		2		2.8	12		15.2	2		3.4	2		0.7	< 0.01
**Osteoporosis with fracture**		3		4.2	3		1.3	3		5.2	1		0.7	0.98
**Past use of b/tsDMARD**	**TL**	20		28.2	1		1.3	45		77.6	43		29.7	N.A
	**TA**	14		19.7	53		67.1	0		0.0	22		15.2	
	**TJ**	4		5.6	7		8.9	7		12.1	17		11.7	
	**LA**	6		8.5	0		0.0	0		0.0	5		3.4	
	**LJ**	2		2.8	0		0.0	0		0.0	0		0.0	
	**AJ**	0		0.0	1		1.3	0		0.0	0		0.0	
	**TLA**	20		28.2	0		0.0	0		0.0	24		16.6	
	**TAJ**	0		0.0	12		15.2	0		0.0	4		2.8	
	**TLJ**	1		1.4	2		2.5	6		10.3	11		7.6	
	**TLAJ**	4		5.6	3		3.8	0		0.0	19		13.1	
**vD2T RA**		25		35.2	17		21.5	6		10.3	58		40.0	< 0.01*

*D2T RA* difficult-to-treat rheumatoid arthritis, *b/tsDMARD* targeted synthetic disease-modifying anti-rheumatic drugs, *TNFi* tumour necrosis factor inhibitor, *IL-6R*, interleukin-6 receptor inhibitor, *CTLA4-Ig* cytotoxic T-lymphocyte–associated antigen-4 immunoglobulin, *JAKi* Janus kinase inhibitor, *CDAI* Clinical Disease Activity Index, *HAQ-DI* Health Assessment Questionnaire Disability Index, *DAS* disease activity score, *CRP* C-reactive protein, *ESR* erythrocyte sedimentation rate, *MTX* methotrexate, *GC* glucocorticoid, *NA* not applicable

*p < 0.05

^a^ Prednisolone equivalent dose

### Trajectories of changes in clinical parameters

**Fig. 3:**
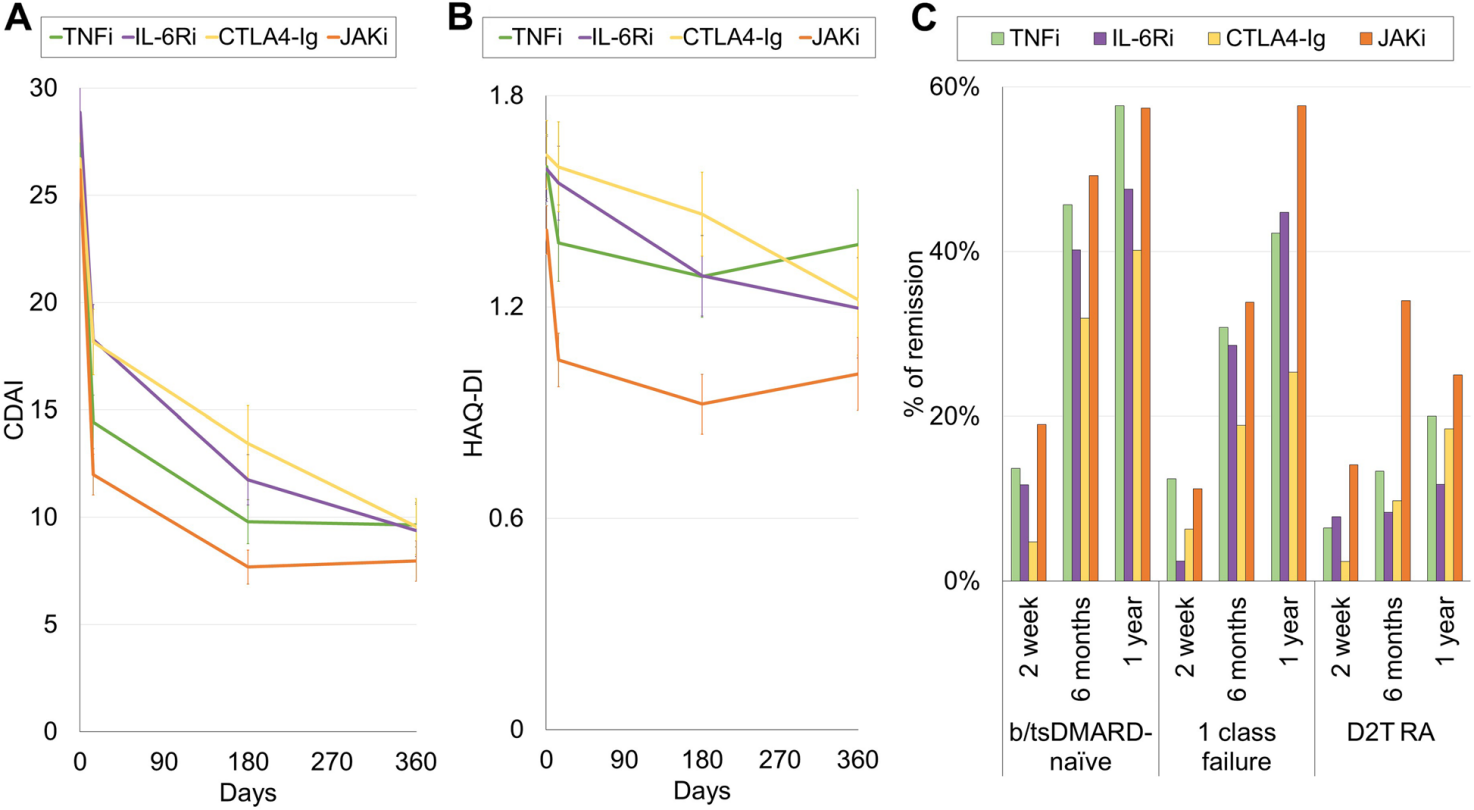
Time trend of clinical parameters. **A** Fluctuation of the average values with standard errors of CDAI in the time trend. **B** Fluctuation of the average values with standard errors of HAQ-DI in the time trend. **C** Time trend of the proportion of remission in the b/tsDMARD-naïve group, the 1 class failure group, and the D2T RA group. CDAI, Clinical Disease Activity Index; HAQ-DI, Health Assessment Questionnaire Disability Index; D2T RA, difficult-to-treat rheumatoid arthritis, b/tsDMARD, targeted synthetic disease-modifying anti-rheumatic drugs; TNFi, tumour necrosis factor inhibitor; IL-6Ri, interleukin-6 receptor inhibitor; CTLA4-Ig, cytotoxic T-lymphocyte-associated antigen-4 immunoglobulin; JAKi, Janus kinase inhibitor

Changes in CDAI and HAQ-DI in cases with D2T RA up to 1 year after the start of the treatment are shown in Fig. 3. The mean CDAI rapidly improved following any of the 4 treatment regimens, but at days 14 and 90, JAKi reduced CDAI to a greater extent than the other 3 regimens. At year 1, CDAI levels were comparable across the 4 regimens (Fig. 3A). The mean HAQ-DI also improved in all of the 4 regimens by year 1 (Fig. 3B). The proportion of remission appeared comparable among the 4 regimens in the b/tsDMARD-naïve group and among those who failed 1 class of b/tsDMARD. On the contrary, the proportion of remission appeared higher among JAKi in D2T RA (Fig. 3C).

**Fig. 4:**
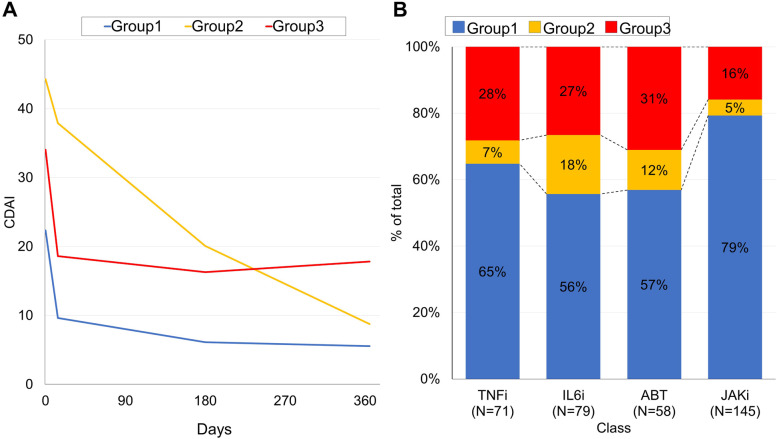
Latent class analysis of patient CDAI responses in D2T RA cases. **A** Three CDAI response trajectories obtained by latent analysis. Group 1, rapid responders; group 2, slow responders; and group 3, less-responders. **B** Percentages of patients included in each group by treatment subgroups. CDAI, Clinical Disease Activity Index; D2T RA, difficult-to-treat rheumatoid arthritis; TNFi, tumour necrosis factor inhibitor; IL-6Ri, interleukin-6 receptor inhibitor; CTLA4-Ig, cytotoxic T-lymphocyte-associated antigen-4 immunoglobulin; JAKi, Janus kinase inhibitor

We previously found that response patterns to b/tsDMARDs can be categorised into 3 groups [7, 9]. Therefore, latent class analysis was conducted for the D2T RA group to categorise the response patterns (Fig. 4A). The first group included rapid responders who had lower baseline CDAI scores, rapidly responded to treatment, and maintained low disease activity for up to 1 year (group 1). The second group included slow responders who had higher baseline CDAI scores and continued to improve after 1 year (group 2). The third group included less responders who showed intermediate disease activity and responded to treatment within 2 weeks but showed no further improvement thereafter (group 3). Proportions of group 1 were higher among the TNFi and JAKi groups, while that of group 3 was lowest in the JAKi group (Fig. 4B). The difference in the proportion of group 1 was significant between IL-6i and JAKi (*p* < 0.01).

### Comparison of treatment effects in D2T RA and vD2T RA cases

It is possible that the aforementioned differences between the 4 subgroups are caused by the difference in the patient background such as age and MTX dose (Table 1). To control for covariates, two types of analyses were conducted. One was a panel analysis using mixed-effects regression. Another was IPTW regression using Δ values after 1 year. In IPTW, factors that showed significance in Table 1 (age, DAS28-ESR, MTX dose, and serum creatinine level) were used for the adjustment.

**Fig. 5:**
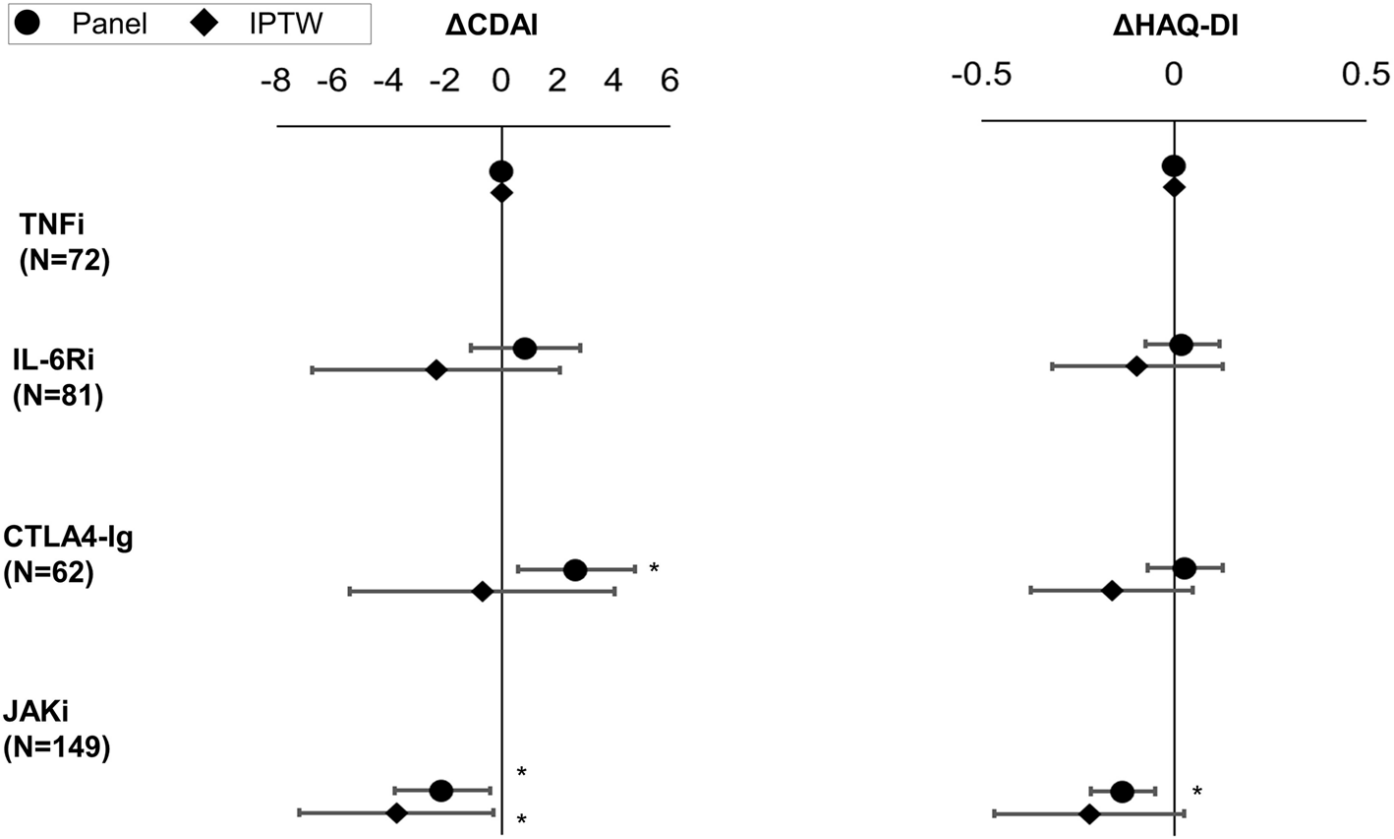
Comparison of treatment outcomes of D2T RA by treatment types. Changes in CDAI and HAQ-DI over the course of a year are compared using panel data analysis and IPTW among D2T RA. TNFi is treated as the reference. *p < 0.05. CDAI, Clinical Disease Activity Index; HAQ-DI, Health Assessment Questionnaire Disability Index; IPTW, propensity-based inverse-probability treatment weighted; D2T RA, difficult-to-treat rheumatoid arthritis; TNFi, tumour necrosis factor inhibitor; IL-6Ri, interleukin-6 receptor inhibitor; CTLA4-Ig, cytotoxic T-lymphocyte–associated antigen-4 immunoglobulin; JAKi, Janus kinase inhibitor

The adjusted patient characteristics are shown in Table 2. Among D2T RA (Fig. 5 and Additional file 3), the JAKi subgroup showed more improvement compared with the TNFi subgroups in CDAI by both panel analysis (coefficient − 2.10, 95% confidence interval [− 3.79, − 0.41], *p* = 0.02) and IPTW (average treatment effect [ATE] − 3.73 [− 716, − 0.29], *p* = 0.03). HAQ-DI was found to be significantly improved among JAKi compared to TNFi by panel analysis (coefficient − 0.13 [− 0.22, − 0.05], *p* < 0.01), but not significant by IPTW (ATE − 0.22 [− 0.47, 0.03], *p* = 0.08). CTLA4-Ig showed less improvement in CDAI compared to TNFi by panel analysis (coefficient 2.66 [0.58, 4.75], *p* = 0.01), but not by IPTW (ATE − 0.68 [− 5.27, 4.01], *p* = 0.78).

**Table 2:** Background of the D2T RA cases after IPTW adjustment, by treatment types

Numerical variable	TNFi (***N*** = 71)		IL6-Ri (***N*** = 78)		CTLA4-Ig (***N*** = 58)		JAKi (***N*** = 143)		***p***
	Mean	SE	Mean	SE	Mean	SE	Mean	SE	
**Age**	64.5	20.5	68.5	14.8	65.5	19.3	62.2	20.3	0.51
**Disease duration (months)**	169.1	135.7	171.7	129.3	191.8	152.7	167.5	131.5	0.18
**CDAI**	28.4	16.0	30.3	18.8	27.6	14.4	27.5	16.5	0.67
**HAQ-DI**	1.6	0.9	1.6	1.0	1.7	0.9	1.5	1.0	0.46
**Dose of MTX (mg/week)**	5.0	1.8	5.1	2.0	4.7	1.6	4.7	2.0	0.50
**Dose of GC (mg/day)**^**a**^	5.8	2.0	6.0	2.1	5.4	2.0	5.5	2.2	0.19
**Serum creatinine (mg/dL)**	0.9	0.7	0.9	0.8	0.7	0.4	0.7	0.4	0.10

*D2T RA* difficult-to-treat rheumatoid arthritis, *IPTW* propensity-based inverse-probability treatment weighted, *SE* standard error, *TNFi* tumour necrosis factor inhibitor, *IL-6Ri* interleukin-6 receptor inhibitor, *CTLA4-Ig* cytotoxic T-lymphocyte–associated antigen-4 immunoglobulin, *JAKi* Janus kinase inhibitor, *CDAI* Clinical Disease Activity Index, *HAQ-DI* Health Assessment Questionnaire Disability Index, *CRP* C-reactive protein, *ESR* erythrocyte sedimentation rate, *DAS* disease activity score, *MTX* methotrexate, *GC* glucocorticoid, *ACPA* anti-cyclic citrullinated peptide antibody

**Fig. 6:**
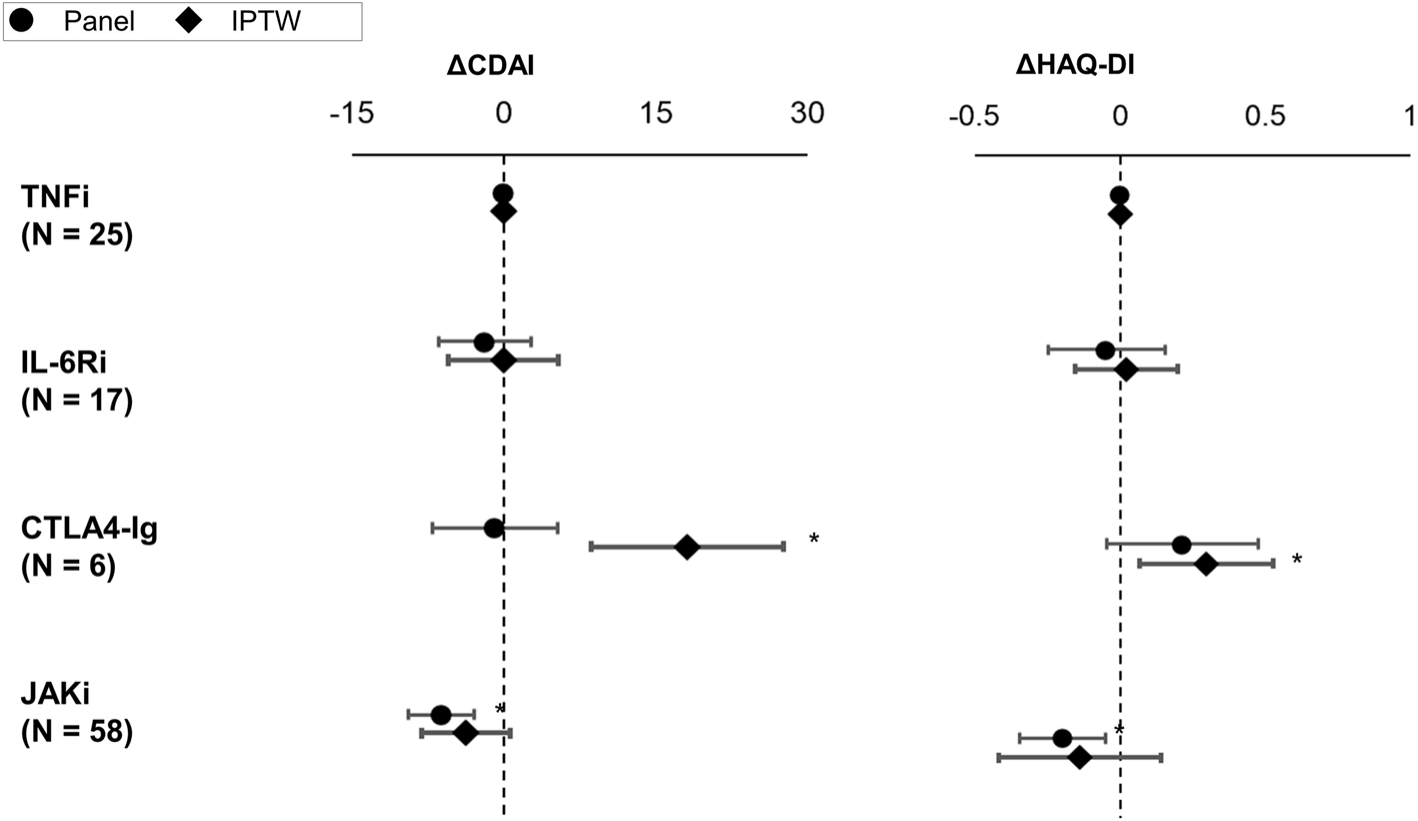
Comparison of treatment outcomes of vD2T RA by treatment types. Change in CDAI and HAQ-DI over the course of a year was compared using panel data analysis and IPTW among vD2T RA. TNFi is treated as a reference. *p < 0.05. vD2T RA, very difficult-to-treat rheumatoid arthritis; CDAI, Clinical Disease Activity Index; HAQ-DI, Health Assessment Questionnaire Disability Index; IPTW, propensity-based inverse-probability treatment weighted; TNFi, tumour necrosis factor inhibitor; IL-6Ri, interleukin-6 receptor inhibitor; CTLA4-Ig, cytotoxic T-lymphocyte-associated antigen-4 immunoglobulin; JAKi, Janus kinase inhibitor

The same analysis was conducted among the vD2T RA group (Fig. 6 and Additional file 4). A significant improvement in CDAI in JAKi compared with TNFi subgroup was observed both by panel analysis (coefficient − 6.21 [− 9.52, − 2.90], *p* < 0.01) but not by IPTW (ATE − 3.73 [− 8.23, 0.65], *p* = 0.10). JAKi also showed significant improvement in HAQ-DI by panel analysis (coefficient − 0.20 [− 0.34, − 0.05], *p* = 0.01) but not by IPTW (ATE − 0.14 [− 0.42, 0.14], *p* = 0.34). The CTLA4-Ig subgroup showed significantly less improvement in HAQ-DI by IPTW (ATE 0.30 [0.07, 0.53], *p* = 0.01) compared with the TNFi subgroup. There was no significant difference between TNFi and IL-6Ri subgroups on any measures in the vD2T RA group.

### Impact of the use of MTX and glucocorticoids

**Fig. 7:**
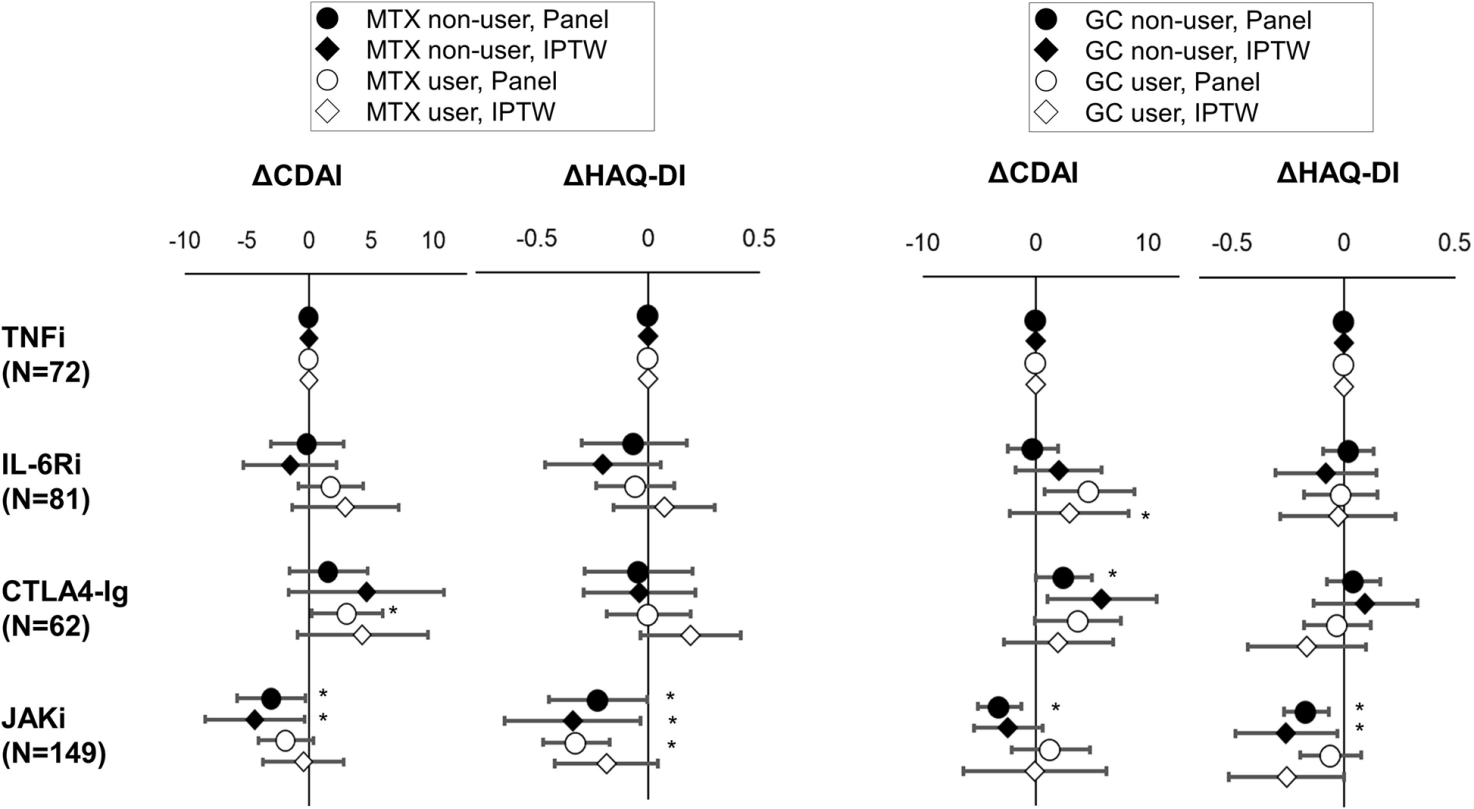
Comparison of treatment outcomes of D2T RA with or without methotrexate and glucocorticoid. Changes in CDAI and HAQ-DI over the course of a year are compared with or without methotrexate and glucocorticoid using panel data analysis and IPTW among D2T RA. TNFi is treated as the reference. *p < 0.05. CDAI, Clinical Disease Activity Index; HAQ-DI, Health Assessment Questionnaire Disability Index; IPTW, propensity-based inverse-probability treatment weighted; D2T RA, difficult-to-treat rheumatoid arthritis; TNFi, tumour necrosis factor inhibitor; IL-6Ri, interleukin-6 receptor inhibitor; CTLA4-Ig, cytotoxic T-lymphocyte–associated antigen-4 immunoglobulin; JAKi, Janus kinase inhibitor

The effectiveness of different classes of b/tsDMARDs might differ based on the concomitant use of MTX or glucocorticoids. Therefore, the same analyses from Fig. 5 were conducted separately for users and non-users of MTX and glucocorticoids (Fig. 7 and Additional file 5). Interestingly, a significant CDAI improvement was observed in the JAKi subgroup compared with the TNFi subgroup in MTX non-users (coefficient − 3.00 [− 5.74, − 0.26], *p* = 0.03; ATE − 4.34 [− 8.34, − 0.35], *p* = 0.03), but not in MTX users (coefficient − 1.83 [− 4.03, 0.37], *p* = 0.10; ATE − 0.44 [− 3.71, 2.83], *p* = 0.79). Significant difference between TNFi and JAKi was observed only in panel analysis of glucocorticoid non-users (coefficient − 3.19 [− 5.15, − 1.23], *p* < 0.01) and not in IPTW (ATE − 2.42 [− 5.49, − 0.66], *p* = 0.12).

A significant improvement in HAQ-DI in the JAKi subgroup was observed in MTX non-users by both panel analysis (coefficient − 0.32 [− 0.47, − 0.17], *p* < 0.01) and IPTW (ATE − 0.34 [− 0.64, − 0.03], *p* = 0.03).

### Hazards of SAEs

**Fig. 8:**
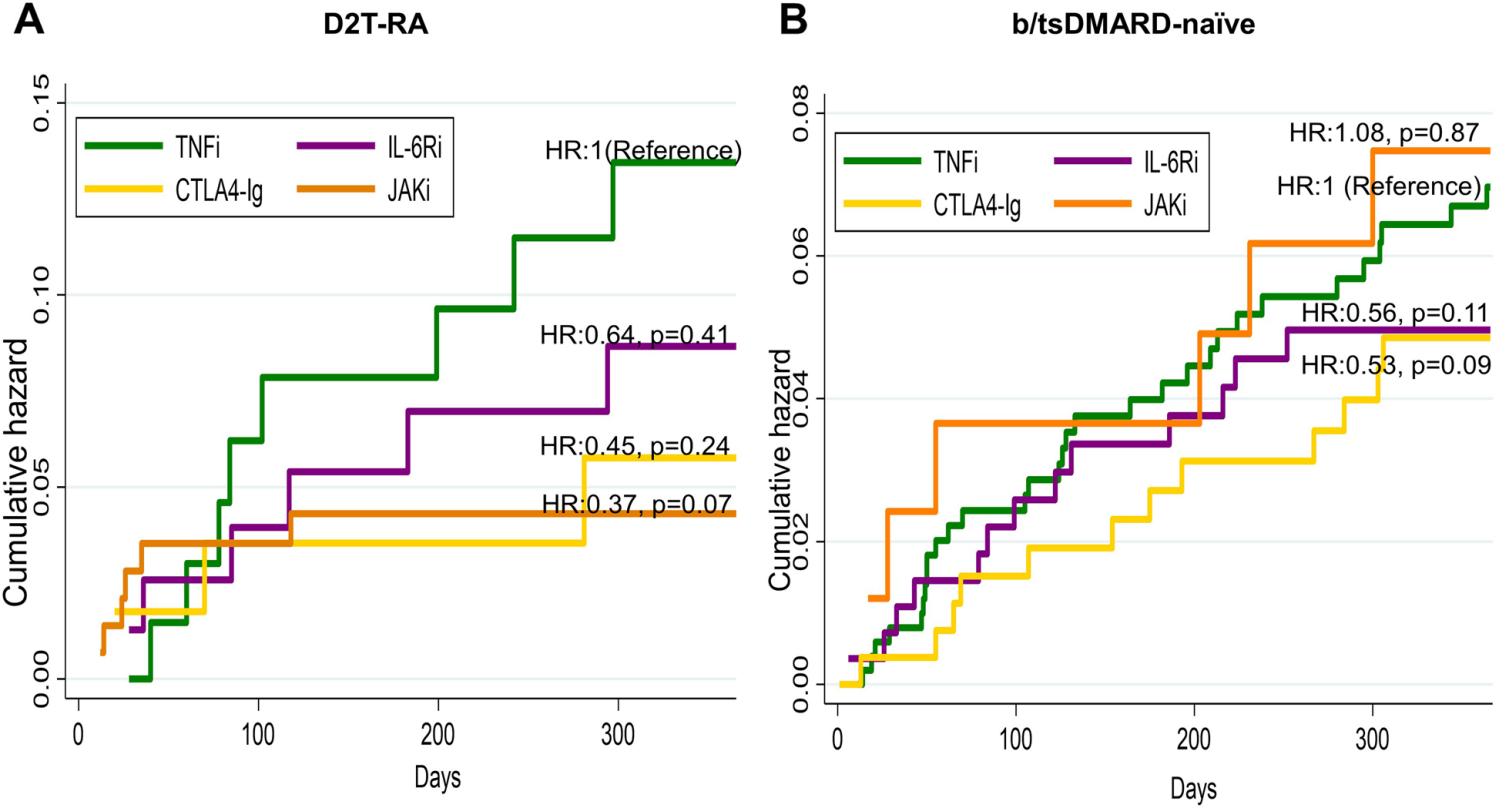
Comparison of hazards of severer adverse event by treatment types. **A** Comparison among the D2T RA group. **B** Comparison among the b/tsDMARD-naïve group. TNFi was used as the reference. Nelson-Aalen cumulative hazard estimate was used. The result of Cox regression using TNFi was used as the reference. *p < 0.05. TNFi, tumour necrosis factor inhibitor; IL-6Ri, interleukin-6 receptor inhibitor; CTLA4-Ig, cytotoxic T-lymphocyte–associated antigen-4 immunoglobulin; JAKi, Janus kinase inhibitor; HR, hazard ratio

Cumulative hazard estimates with calculated hazard ratio (HR) are shown in Fig. 8 and Additional file 6.

In total, 33 D2T RA cases (TNFi 11, IL-6Ri 7, CTLA4-Ig 5, JAKi 10) and 73 b/tsDMARD-naïve cases (TNFi 34, IL-6Ri 17, CTLA4-Ig 15, and JAKi 7) stopped treatment due to SAEs over the course of a year, most of which were adverse reaction. The breakdown of SAE is shown in Additional file 7. After controlling for background variables, no difference in the risk of SAEs was observed in the both D2T RA and b/tsDMARD-naïve groups.

## Discussion

This study is unique in that it compared responsiveness to different classes of b/tsDMARDs among D2T RA and vD2T RA cases who were refractory to multiple b/tsDMARDs. Of the 4 b/tsDMARDs with different mechanisms of action studied, JAKis were associated with the highest proportion of rapid responders and the best outcomes in CDAI. This effect was highlighted in MTX- and glucocorticoid-free individuals.

There are several studies targeting RA patients who had failed treatment with ≥ 2 b/tsDMARDs. Favourable treatment outcomes have been reported for such patients with baricitinib, upadacitinib, and filgotinib compared with placebo [11–17]. Other studies on patients who had failed two TNFi treatments showed favourable outcomes with mavrilimumab and rituximab compared with alternative TNFis [18–20]. However, these studies often include patients who were refractory to only one b/tsDMARD or two b/tsDMARDs with the same mechanism of action, which are not classified as D2T RA according to the new EULAR definition. In addition, none of these studies compared multiple classes of b/tsDMARDs. A systematic review compared the results of clinical trials of different b/tsDMARDs [21], but it included only one study that conducted a head-to-head comparison [22]. Our study has the strength that it compared 4 classes of b/tsDMARDs in the same cohort during the same period.

Better outcomes by JAKis compared to other classes of b/tsDMARDs have been implicated in a systematic review [21], which showed that JAKi treatment had the highest probability of ACR50 achievement compared with other b/tsDMARDs. Our findings are consistent with that result. Our analysis (Fig. 3) suggested that this effectiveness of JAKis can partly be explained by the small proportion of less-responders, presumably due to their broad action [23, 24]. RA is associated with the overproduction of a variety of cytokines in addition to TNF and IL-6, such as IL-12, IL-15, IL-23, granulocyte-macrophage colony-stimulating factor, and interferons. As the JAK/signal transducer and activator of transcription (STAT) pathway are used by receptors for many of these cytokines, JAKis may exert effectiveness on D2T RA by inhibiting a wide range of cytokines. Some patients also develop anti-drug antibodies (ADA) against multiple b/tsDMARDs [25, 26], which can cause D2T RA. As JAKis reduce the immunogenicity of drugs [27], the production of ADAs can be reduced by treatment with JAKi. This mechanism may explain the superiority of JAKis among non-users of MTX and glucocorticoid, which also act to prevent the generation of ADAs [28]. Further research is required to elucidate the molecular mechanisms of refractoriness and effectiveness of each b/tsDMARD in D2T RA patients.

This study also showed that risks of SAE were comparable between the treatment subgroups, though allergic reactions among TNFi subgroups appeared to be higher than other subgroups (Table S6). As such, reactions are rare among the JAKi subgroups, switching to JAKi might be a preferable option for the next treatment if a D2T RA status is caused by infusion reactions. Although cardiovascular events were a major concern among RA patients [29], only one case of cardiovascular event that led to treatment cessation was observed in our study. This may support previous findings of reduced risks of cardiovascular events among b/tsDMARD users [30], though this may also due to the short observational period of 1 year.

However, even when JAKis appear to be a preferable option for a group of D2T RA patients with regard to CDAI, it does not mean JAKis should be recommended to all D2T RA patients. D2T RA comprises a variety of concepts that were not fully included in our study, such as comorbidities that limit treatment choice and noninflammatory pain [31]. For example, CTLA4-Ig was reported to have fewer adverse effects on interstitial lung diseases than other bDMARDs [32] and thus is preferred in such types of D2T RA. Other factors such as long-term safety profile, forms of drugs, and factors that affect patients’ adherence to treatment [33] must also be taken into consideration. For example, in the treatment of patients with dementia, non-oral drugs might be preferred to oral medications [34, 35]. Even so, our results at least suggest that JAKi might be considered for treatment choice for patients who are refractory to multiple treatments.

Our study has several limitations, primarily due to its retrospective nature. First, this registry included several episodes of treatments from the same patients who received different agents. Second, the registry collected names of coexisting diseases, but the severity of each disease was not recorded. Therefore, although this study included factors related to some comorbidities, namely, the coexistence of chronic lung diseases and serum titres of creatinine, there may be other comorbidities confounding the outcomes such as hepatic disorders and neurological disorders. In addition, our data does not include non-inflammatory symptoms such as fibromyalgia, which are included as a major criterion of the EULAR definition [3] as well as a point to consider in the management of D2T RA [4]. The number of past fractures was also small, suggesting our data about comorbidities was insufficient. From this limitation, even though multiple regression and IPTW were used, bias was not removed entirely. Third, some treatment options such as rituximab were not included in our study because they have not been approved as an RA treatment in Japan. In addition, due to the longer history of TNFi compared to other drugs, most of the D2T RA cases were former TNFi users, which may skew the effectiveness of TNFis to the D2T RA cases. However, even with these limitations, our study is important in that it provides an important evidence for optimal treatment for D2T RA and vD2T RA patients.

## Conclusions

This study suggests the effectiveness of JAKi for a subgroup of D2T RA and vD2T RA patients, especially when the patients were not treated with glucocorticoid or MTX. Further research is required to elucidate the molecular mechanism of refractoriness and effectiveness of each b/tsDMARD among D2T RA patients.

## Supplementary Information

**Additional file 1:** **Table S1.** Breakdown of underlying diseases that required high dose of corticosteroid.

**Additional file 2:** **Figure S1.** Patterns of the treatment switches in D2T-RA patients.

**Additional file 3:** **Table S2.** Comparison of treatment outcomes of D2T-RA by treatment types. D2T-RA, difficult-to-treat rheumatoid arthritis; IPTW, propensity-based inverse-probability treatment weighted; ATE, average treatment effect; CI, confidence interval; CDAI, clinical disease activity index; HAQ-DI, health assessment questionnaire disability index; TNFi, tumour necrosis factor inhibitor; IL-6Ri, interleukin-6 receptor inhibitor; CTLA4-Ig, cytotoxic T-lymphocyte–associated antigen-4 immunoglobulin; JAKi, Janus kinase inhibitor. * *p*<0.05.

**Additional file 4:** **Table S3.** Comparison of treatment outcomes of vD2T-RA by treatment types. vD2T-RA, very difficult-to-treat RA; IPTW, propensity-based inverse-probability treatment weighted; ATE, average treatment effect; CI, confidence interval; CDAI, clinical disease activity index; HAQ-DI, health assessment questionnaire disability index; TNFi, tumour necrosis factor inhibitor; IL-6Ri, interleukin-6 receptor inhibitor; CTLA4-Ig, cytotoxic T-lymphocyte–associated antigen-4 immunoglobulin; JAKi, Janus kinase inhibitor. * *p*<0.05.

**Additional file 5:** **Table S4.** Comparison of treatment outcomes of D2T-RA with or without methotrexate and glucocorticoid. D2T-RA, difficult-to-treat rheumatoid arthritis; MTX, methotrexate; GC, glucocorticoid; IPTW, propensity-based inverse-probability treatment weighted; Coeff, coefficient; ATE, average treatment effect; CI, confidence interval; CDAI, clinical disease activity index; HAQ-DI, health assessment questionnaire disability index; TNFi, tumour necrosis factor inhibitor; IL-6Ri, interleukin-6 receptor inhibitor; CTLA4-Ig, cytotoxic T-lymphocyte–associated antigen-4 immunoglobulin; JAKi, Janus kinase inhibitor. * *p*<0.05.

**Additional file 6:** **Table S5.** Comparison of hazards of severe adverse events by treatment types. Cox regression analysis was conducted controlling for age, gender, dose of methotrexate and glucocorticoid at day 0. D2T-RA, difficult-to-treat rheumatoid arthritis; b/tsDMARD, targeted synthetic disease-modifying anti-rheumatic drugs; HR, hazard ratio; CI, confidence interval; TNFi, tumour necrosis factor inhibitor; IL-6Ri, interleukin-6 receptor inhibitor; CTLA4-Ig, cytotoxic T-lymphocyte–associated antigen-4 immunoglobulin; JAKi, Janus kinase inhibitor. *p<0.05.

**Additional file 7:** **Table S6.** Breakdown of severe adverse events in the D2T-RA and b/tsDMARD-naïve groups. D2T-RA, difficult-to-treat rheumatoid arthritis; b/tsDMARD, targeted synthetic disease-modifying anti-rheumatic drugs; I TNFi, tumour necrosis factor inhibitor; IL-6Ri,interleukin-6 receptor inhibitor; CTLA4-Ig, cytotoxic T-lymphocyte–associated antigen-4 immunoglobulin; JAKi, Janus kinase inhibitor; LPD, Lymphoproliferative disorder; CHF: congestive heart disease; CK, creatinine kinase; LOC: loss of consciousness.

## Data Availability

The datasets used and/or analysed during the current study are available from the corresponding author on reasonable request.

## Abbreviations

ACPA: Anti-cyclic citrullinated protein antibody
b/tsDMARD: Biologic and targeted synthetic disease-modifying anti-rheumatic drug
CDAI: Clinical Disease Activity Index
CRP: C-reactive protein
CTLA-Ig: Cytotoxic T-lymphocyte–associated antigen-4 immunoglobulin
DAS: Disease activity score
D2T RA: Difficult-to-treat rheumatoid arthritis
ESR: Erythrocyte sedimentation rate
EULAR: European Alliance of Associations for Rheumatology
GC: Glucocorticoid
HAQ-DI: Health Assessment Questionnaire Disability Index
HR: Hazard ratio
IL-6i: Interleukin-6 receptor inhibitor
IPTW: Inverse-probability treatment weighted
JAKi: Janus-kinase inhibitor
MTX: Methotrexate
RA: Rheumatoid arthritis
SAE: Severe adverse event
TNF: Tumour necrosis factor inhibitor
vD2T RA: Very difficult-to-treat rheumatoid arthritis
