# Supplementary material for: Preferable outcome of Janus kinase inhibitors for a group of difficult-to-treat rheumatoid arthritis patients: from the FIRST Registry
Source: Arthritis Res Ther. 2022 Mar 1;24:61. doi: 10.1186/s13075-022-02744-7 (PMC8886884; doi:10.1186/s13075-022-02744-7)
Supplement: Supplementary file 1 — Additional file 1: Table S1. Breakdown of underlying diseases that required high dose of corticosteroid. [file 13075_2022_2744_MOESM1_ESM.docx]

**Additional file 1.** **Breakdown of underlying diseases that required high dose of corticosteroid.**

| Rheumatoid arthritis with vasculitis | 19 |
| --- | --- |
| Interstitial pneumonia | 13 |
| Inflammatory bowel disease | 3 |
| Sarcoidosis | 2 |
| Autoimmune hepatitis | 2 |
| IgG4-related disease | 1 |
| Systemic lupus erythematosus | 1 |
| Myositis | 1 |
| Other autoimmune diseases | 9 |
| Eosinophilic pneumonia | 1 |
| Emphysema | 1 |
| Pyoderma gangrenosum | 1 |
| Psoriasis | 1 |
| Relapsing polychondritis | 1 |
| Nephritis | 1 |
| Other lung diseases | 5 |
| Other | 6 |
