# Supplementary figures and images for: Preferable outcome of Janus kinase inhibitors for a group of difficult-to-treat rheumatoid arthritis patients: from the FIRST Registry
Source: Arthritis Res Ther. 2022 Mar 1;24:61. doi: 10.1186/s13075-022-02744-7 (PMC8886884; doi:10.1186/s13075-022-02744-7)

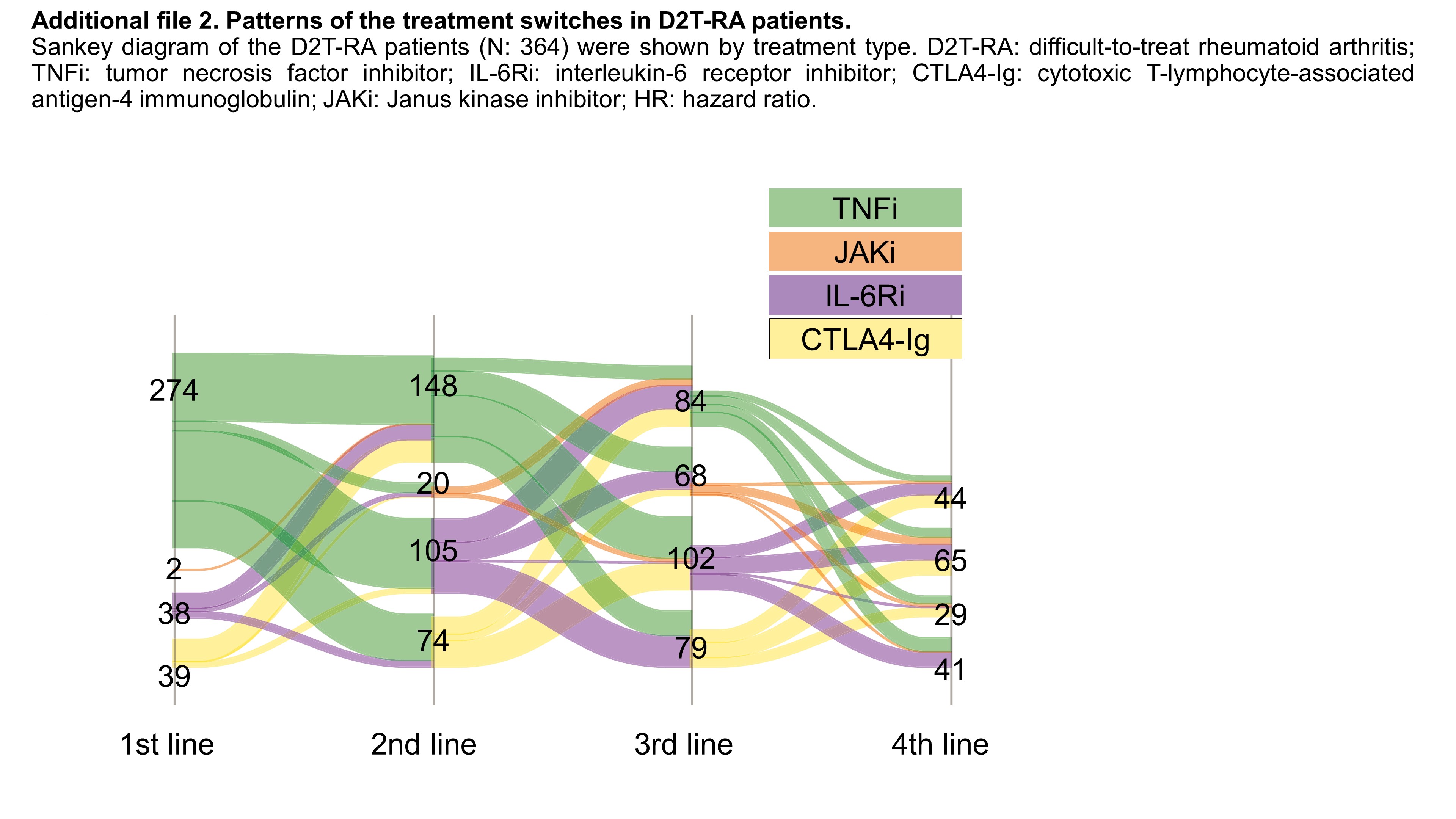

Supplement: Supplementary file 2 — Additional file 2: Figure S1. Patterns of the treatment switches in D2T-RA patients. [file 13075_2022_2744_MOESM2_ESM.jpg]
