# Supplementary material for: Preferable outcome of Janus kinase inhibitors for a group of difficult-to-treat rheumatoid arthritis patients: from the FIRST Registry
Source: Arthritis Res Ther. 2022 Mar 1;24:61. doi: 10.1186/s13075-022-02744-7 (PMC8886884; doi:10.1186/s13075-022-02744-7)
Supplement: Supplementary file 3 — Additional file 3: Table S2. Comparison of treatment outcomes of D2T-RA by treatment types. D2T-RA, difficult-to-treat rheumatoid arthritis; IPTW, propensity-based inverse-probability treatment weighted; ATE, average treatment effect; CI, confidence interval; CDAI, clinical disease activity index; HAQ-DI, health assessment questionnaire disability index; TNFi, tumour necrosis factor inhibitor; IL-6Ri, interleukin-6 receptor inhibitor; CTLA4-Ig, cytotoxic T-lymphocyte–associated antigen-4 immunoglobulin; JAKi, Janus kinase inhibitor. * p<0.05. [file 13075_2022_2744_MOESM3_ESM.docx]

**Additional file 3. Comparison of treatment outcomes of D2T-RA by treatment types.**

|  | | **Panel analysis** | | | | **IPTW** | | | |
| --- | --- | --- | --- | --- | --- | --- | --- | --- | --- |
|  |  | **Coefficient** | **95% CI** | | **p** | **ATE** | **95% CI** | | **p** |
| **CDAI** | **TNFi** | 0 (Reference) | | | | 0 (Reference) | | | |
|  | **IL-6Ri** | 0.85 | -1.09 | 2.79 | 0.39 | -2.32 | -6.72 | 2.07 | 0.30 |
|  | **CTLA4-Ig** | 2.66 | 0.58 | 4.75 | 0.01* | -0.68 | -5.37 | 4.01 | 0.78 |
|  | **JAKi** | -2.10 | -3.79 | -0.41 | 0.02* | -3.73 | -7.16 | -0.29 | 0.03* |
| **HAQ-DI** | **TNFi** | 0 (Reference) | | | | 0 (Reference) | | | |
|  | **IL-6Ri** | 0.02 | -0.07 | 0.12 | 0.66 | -0.10 | -0.32 | 0.13 | 0.40 |
|  | **CTLA4-Ig** | 0.03 | -0.07 | 0.13 | 0.55 | -0.16 | -0.37 | 0.05 | 0.13 |
|  | **JAKi** | -0.13 | -0.22 | -0.05 | <0.01* | -0.22 | -0.47 | 0.03 | 0.08 |

D2T-RA, difficult-to-treat rheumatoid arthritis; IPTW, propensity-based inverse-probability treatment weighted; ATE, average treatment effect; CI, confidence interval; CDAI, clinical disease activity index; HAQ-DI, health assessment questionnaire disability index; TNFi, tumor necrosis factor inhibitor; IL-6Ri, interleukin-6 receptor inhibitor; CTLA4-Ig, cytotoxic T-lymphocyte–associated antigen-4 immunoglobulin; JAKi, Janus kinase inhibitor. * p<0.05.
