# Supplementary material for: Preferable outcome of Janus kinase inhibitors for a group of difficult-to-treat rheumatoid arthritis patients: from the FIRST Registry
Source: Arthritis Res Ther. 2022 Mar 1;24:61. doi: 10.1186/s13075-022-02744-7 (PMC8886884; doi:10.1186/s13075-022-02744-7)
Supplement: Supplementary file 4 — Additional file 4: Table S3. Comparison of treatment outcomes of vD2T-RA by treatment types. vD2T-RA, very difficult-to-treat RA; IPTW, propensity-based inverse-probability treatment weighted; ATE, average treatment effect; CI, confidence interval; CDAI, clinical disease activity index; HAQ-DI, health assessment questionnaire disability index; TNFi, tumour necrosis factor inhibitor; IL-6Ri, interleukin-6 receptor inhibitor; CTLA4-Ig, cytotoxic T-lymphocyte–associated antigen-4 immunoglobulin; JAKi, Janus kinase inhibitor. * p<0.05. [file 13075_2022_2744_MOESM4_ESM.docx]

**Additional file 4. Comparison of treatment outcomes of vD2T-RA by treatment types.**

|  | | **Panel analysis** | | | | **IPTW** | | | |
| --- | --- | --- | --- | --- | --- | --- | --- | --- | --- |
|  |  | **Coefficient** | **95% CI** | | **p** | **ATE** | **95% CI** | | **p** |
| **CDAI** | **TNFi** | 0 (Reference) | | | | 0 (Reference) | | | |
|  | **IL-6Ri** | -1.87 | -6.41 | 2.67 | 0.42 | -0.05 | -5.48 | 5.38 | 0.99 |
|  | **CTLA4-Ig** | -0.89 | -7.09 | 5.31 | 0.78 | 18.14 | 8.65 | 27.62 | <0.01* |
|  | **JAKi** | -6.21 | -9.52 | -2.90 | <0.01* | -3.73 | -8.12 | 0.65 | 0.10 |
| **HAQ-DI** | **TNFi** | 0 (Reference) | | | | 0 (Reference) | | | |
|  | **IL-6Ri** | -0.05 | -0.25 | 0.15 | 0.65 | 0.02 | -0.15 | 0.20 | 0.81 |
|  | **CTLA4-Ig** | 0.22 | -0.04 | 0.48 | 0.10 | 0.30 | 0.07 | 0.53 | 0.01* |
|  | **JAKi** | -0.20 | -0.34 | -0.05 | 0.01* | -0.14 | -0.42 | 0.14 | 0.34 |

vD2T-RA, very difficult-to-treat RA; IPTW, propensity-based inverse-probability treatment weighted; ATE, average treatment effect; CI, confidence interval; CDAI, clinical disease activity index; HAQ-DI, health assessment questionnaire disability index; TNFi, tumour necrosis factor inhibitor; IL-6Ri, interleukin-6 receptor inhibitor; CTLA4-Ig, cytotoxic T-lymphocyte–associated antigen-4 immunoglobulin; JAKi, Janus kinase inhibitor. * p<0.05.
