# Supplementary material for: Preferable outcome of Janus kinase inhibitors for a group of difficult-to-treat rheumatoid arthritis patients: from the FIRST Registry
Source: Arthritis Res Ther. 2022 Mar 1;24:61. doi: 10.1186/s13075-022-02744-7 (PMC8886884; doi:10.1186/s13075-022-02744-7)
Supplement: Supplementary file 5 — Additional file 5: Table S4. Comparison of treatment outcomes of D2T-RA with or without methotrexate and glucocorticoid. D2T-RA, difficult-to-treat rheumatoid arthritis; MTX, methotrexate; GC, glucocorticoid; IPTW, propensity-based inverse-probability treatment weighted; Coeff, coefficient; ATE, average treatment effect; CI, confidence interval; CDAI, clinical disease activity index; HAQ-DI, health assessment questionnaire disability index; TNFi, tumour necrosis factor inhibitor; IL-6Ri, interleukin-6 receptor inhibitor; CTLA4-Ig, cytotoxic T-lymphocyte–associated antigen-4 immunoglobulin; JAKi, Janus kinase inhibitor. * p<0.05. [file 13075_2022_2744_MOESM5_ESM.docx]

**Additional file 5.** Comparison of treatment outcomes of D2T-RA with or without methotrexate and glucocorticoid.

|  | | | **Panel analysis** | | | | **IPTW** | | | |
| --- | --- | --- | --- | --- | --- | --- | --- | --- | --- | --- |
|  |  |  | **Coeff** | **95% CI** | | **p** | **ATE** | **95% CI** | | **p** |
| **CDAI** | **MTX**  **non-user** | **TNFi** | 0 (Reference) | | | | 0 (Reference) | | | |
|  |  | **IL-6Ri** | -0.14 | -3.07 | 2.79 | 0.92 | -1.51 | -5.30 | 2.27 | 0.43 |
|  |  | **CTLA4-Ig** | 1.62 | -1.53 | 4.78 | 0.31 | 4.65 | -1.61 | 10.91 | 0.15 |
|  |  | **JAKi** | -3.00 | -5.74 | -0.26 | 0.03* | -4.34 | -8.34 | -0.35 | 0.03* |
|  | **MTX**  **user** | **TNFi** | 0 (Reference) | | | | 0 (Reference) | | | |
|  |  | **IL-6Ri** | 1.79 | -0.81 | 4.40 | 0.18 | 2.98 | -1.31 | 7.27 | 0.17 |
|  |  | **CTLA4-Ig** | 3.11 | 0.25 | 5.96 | 0.03* | 4.35 | -0.89 | 9.58 | 0.10 |
|  |  | **JAKi** | -1.83 | -4.03 | 0.37 | 0.10 | -0.44 | -3.71 | 2.83 | 0.79 |
| **HAQ-DI** | **MTX**  **non-user** | **TNFi** | 0 (Reference) | | | | 0 (Reference) | | | |
|  |  | **IL-6Ri** | -0.06 | -0.30 | 0.17 | 0.60 | -0.20 | -0.46 | 0.06 | 0.13 |
|  |  | **CTLA4-Ig** | -0.04 | -0.28 | 0.20 | 0.73 | -0.04 | -0.29 | 0.21 | 0.75 |
|  |  | **JAKi** | -0.22 | -0.44 | 0.00 | 0.05* | -0.34 | -0.64 | -0.03 | 0.03* |
|  | **MTX**  **user** | **TNFi** | 0 (Reference) | | | | 0 (Reference) | | | |
|  |  | **IL-6Ri** | -0.06 | -0.23 | 0.12 | 0.53 | 0.07 | -0.16 | 0.30 | 0.54 |
|  |  | **CTLA4-Ig** | 0.00 | -0.18 | 0.19 | 0.98 | 0.19 | -0.03 | 0.41 | 0.09 |
|  |  | **JAKi** | -0.32 | -0.47 | -0.17 | <0.01* | -0.19 | -0.42 | 0.04 | 0.11 |
| **CDAI** | **GC**  **non-user** | **TNFi** | 0 (Reference) | | | | 0 (Reference) | | | |
|  |  | **IL-6Ri** | -0.25 | -2.49 | 1.99 | 0.83 | 2.07 | -1.74 | 5.88 | 0.29 |
|  |  | **CTLA4-Ig** | 2.53 | 0.02 | 5.03 | 0.05* | 5.90 | 1.02 | 10.79 | 0.02* |
|  |  | **JAKi** | -3.19 | -5.15 | -1.23 | <0.01* | -2.42 | -5.49 | 0.66 | 0.12 |
|  | **GC**  **user** | **TNFi** | 0 (Reference) | | | | 0 (Reference) | | | |
|  |  | **IL-6Ri** | 4.79 | 0.76 | 8.81 | 0.02* | 2.99 | -2.26 | 8.24 | 0.26 |
|  |  | **CTLA4-Ig** | 3.78 | -0.03 | 7.58 | 0.05 | 2.04 | -2.84 | 6.91 | 0.41 |
|  |  | **JAKi** | 1.35 | -2.10 | 4.81 | 0.44 | -0.07 | -6.43 | 6.29 | 0.98 |
| **HAQ-DI** | **GC**  **non-user** | **TNFi** | 0 (Reference) | | | | 0 (Reference) | | | |
|  |  | **IL-6Ri** | 0.02 | -0.09 | 0.14 | 0.71 | -0.08 | -0.31 | 0.15 | 0.49 |
|  |  | **CTLA4-Ig** | 0.04 | -0.08 | 0.17 | 0.48 | 0.10 | -0.14 | 0.33 | 0.41 |
|  |  | **JAKi** | -0.17 | -0.27 | -0.07 | <0.01* | -0.26 | -0.49 | -0.03 | 0.03* |
|  | **GC**  **user** | **TNFi** | 0 (Reference) | | | | 0 (Reference) | | | |
|  |  | **IL-6Ri** | -0.01 | -0.18 | 0.15 | 0.88 | -0.03 | -0.28 | 0.23 | 0.85 |
|  |  | **CTLA4-Ig** | -0.03 | -0.18 | 0.12 | 0.72 | -0.17 | -0.43 | 0.10 | 0.22 |
|  |  | **JAKi** | -0.06 | -0.20 | 0.08 | 0.41 | -0.26 | -0.51 | 0.00 | 0.05 |

D2T-RA,difficult-to-treat rheumatoid arthritis; MTX, methotrexate; GC, glucocorticoid; IPTW, propensity-based inverse-probability treatment weighted; Coeff, coefficient; ATE, average treatment effect; CI, confidence interval; CDAI, clinical disease activity index; HAQ-DI, health assessment questionnaire disability index; TNFi, tumour necrosis factor inhibitor; IL-6Ri,interleukin-6 receptor inhibitor; CTLA4-Ig,cytotoxic T-lymphocyte–associated antigen-4 immunoglobulin; JAKi, Janus kinase inhibitor. * p<0.05.
