# Supplementary material for: Preferable outcome of Janus kinase inhibitors for a group of difficult-to-treat rheumatoid arthritis patients: from the FIRST Registry
Source: Arthritis Res Ther. 2022 Mar 1;24:61. doi: 10.1186/s13075-022-02744-7 (PMC8886884; doi:10.1186/s13075-022-02744-7)
Supplement: Supplementary file 6 — Additional file 6: Table S5. Comparison of hazards of severe adverse events by treatment types. Cox regression analysis was conducted controlling for age, gender, dose of methotrexate and glucocorticoid at day 0. D2T-RA, difficult-to-treat rheumatoid arthritis; b/tsDMARD, targeted synthetic disease-modifying anti-rheumatic drugs; HR, hazard ratio; CI, confidence interval; TNFi, tumour necrosis factor inhibitor; IL-6Ri, interleukin-6 receptor inhibitor; CTLA4-Ig, cytotoxic T-lymphocyte–associated antigen-4 immunoglobulin; JAKi, Janus kinase inhibitor. *p<0.05. [file 13075_2022_2744_MOESM6_ESM.docx]

**Additional file 6.** **Comparison of hazards of severe adverse events by treatment types.** Cox regression analysis was conducted controlling for age, gender, dose of methotrexate and glucocorticoid at day 0.

|  | | **HR** | **95% CI** | | **p** |
| --- | --- | --- | --- | --- | --- |
| **D2T-RA** | **TNFi** | 1(Reference) | | |  |
|  | **IL-6Ri** | 0.64 | 0.22 | 1.87 | 0.41 |
|  | **CTLA4-Ig** | 0.45 | 0.12 | 1.71 | 0.24 |
|  | **JAKi** | 0.37 | 0.13 | 1.08 | 0.07 |
| **b/tsDMARD-naïve** | **TNFi** | 1(Reference) | | |  |
|  | **IL-6Ri** | 0.56 | 0.27 | 1.14 | 0.11 |
|  | **CTLA4-Ig** | 0.53 | 0.25 | 1.11 | 0.09 |
|  | **JAKi** | 1.08 | 0.45 | 2.60 | 0.87 |

D2T-RA, difficult-to-treat rheumatoid arthritis; b/tsDMARD, targeted synthetic disease-modifying anti-rheumatic drugs; HR, hazard ratio; CI, confidence interval; TNFi, tumour necrosis factor inhibitor; IL-6Ri, interleukin-6 receptor inhibitor; CTLA4-Ig, cytotoxic T-lymphocyte–associated antigen-4 immunoglobulin; JAKi, Janus kinase inhibitor. *p<0.05.
