# Supplementary material for: Preferable outcome of Janus kinase inhibitors for a group of difficult-to-treat rheumatoid arthritis patients: from the FIRST Registry
Source: Arthritis Res Ther. 2022 Mar 1;24:61. doi: 10.1186/s13075-022-02744-7 (PMC8886884; doi:10.1186/s13075-022-02744-7)
Supplement: Supplementary file 7 — Additional file 7: Table S6. Breakdown of severe adverse events in the D2T-RA and b/tsDMARD-naïve groups. D2T-RA, difficult-to-treat rheumatoid arthritis; b/tsDMARD, targeted synthetic disease-modifying anti-rheumatic drugs; I TNFi, tumour necrosis factor inhibitor; IL-6Ri,interleukin-6 receptor inhibitor; CTLA4-Ig, cytotoxic T-lymphocyte–associated antigen-4 immunoglobulin; JAKi, Janus kinase inhibitor; LPD, Lymphoproliferative disorder; CHF: congestive heart disease; CK, creatinine kinase; LOC: loss of consciousness. [file 13075_2022_2744_MOESM7_ESM.docx]

**Additional file 7. Breakdown of severe adverse events in the D2T-RA and b/tsDMARD-naïve groups.**

|  | **D2T-RA** | | | | **b/tsDMARD-naïve** | | | |
| --- | --- | --- | --- | --- | --- | --- | --- | --- |
|  | **TNFi**  **(N=71)** | **IL-6Ri**  **(N=79)** | **CTLA4-Ig**  **(N=58)** | **JAKi**  **(N=145)** | **TNFi**  **(N=513)** | **IL-6Ri**  **(N=279)** | **CTLA4-Ig**  **(N=268)** | **JAKi**  **(N=83)** |
| **Skin rash** | 4 | 0 | 2 | 0 | 8 | 5 | 0 | 1 |
| **Infusion reaction** | 2 | 1 | 0 | 0 | 11 | 0 | 2 | 0 |
| **Other allergy** | 2 | 0 | 0 | 0 |  | 2 | 0 | 0 |
| **Infection** | 1 | 0 | 0 | 2 | 3 | 1 | 2 | 1 |
| **Drug-induced pneumonia** | 0 | 0 | 0 | 0 | 0 | 0 | 0 | 0 |
| **LPD** | 0 | 1 | 0 | 1 | 2 | 2 | 0 | 0 |
| **Malignancy** | 0 | 0 | 1 | 0 | 0 | 0 | 0 | 0 |
| **Aortitis** | 1 | 0 | 0 | 0 | 0 | 0 | 1 | 0 |
| **Asthma** | 0 | 0 | 0 | 0 | 0 | 0 | 1 | 0 |
| **CHF** | 0 | 0 | 0 | 0 | 0 | 0 | 1 | 0 |
| **Depression** | 0 | 0 | 0 | 0 | 0 | 0 | 1 | 0 |
| **Diarrhoea** | 0 | 0 | 0 | 1 | 0 | 2 | 0 | 0 |
| **Dizziness** | 0 | 0 | 0 | 1 | 0 | 0 | 0 | 0 |
| **Drowsiness** | 0 | 0 | 0 | 0 | 0 | 0 | 0 | 1 |
| **Dry mouth** | 0 | 0 | 0 | 0 | 0 | 1 | 0 | 0 |
| **Dysgeusia** | 0 | 0 | 0 | 0 | 0 | 0 | 0 | 1 |
| **Elevation of CK** | 0 | 0 | 0 | 0 | 0 | 0 | 0 | 1 |
| **Emphysema** | 0 | 0 | 0 | 0 | 0 | 0 | 1 | 0 |
| **Fatigue** | 0 | 0 | 0 | 0 | 2 | 0 | 0 | 1 |
| **Fever** | 0 | 1 | 0 | 0 | 0 | 0 | 0 | 0 |
| **Hepatic disorder** | 1 | 0 | 0 | 0 | 2 | 3 | 2 | 0 |
| **Indefinite complaint** | 0 | 0 | 1 | 0 | 0 | 0 | 0 | 0 |
| **Leukopenia** | 0 | 1 | 0 | 0 | 0 | 0 | 0 | 0 |
| **LOC** | 0 | 0 | 0 | 0 | 1 | 0 | 0 | 0 |
| **Lupus-like syndrome** | 0 | 0 | 0 | 0 | 1 | 0 | 0 | 0 |
| **Nausea** | 0 | 0 | 0 | 0 | 1 | 0 | 0 | 1 |
| **Oedema** | 0 | 0 | 0 | 0 | 1 | 0 | 0 | 0 |
| **Oral ulcer** | 0 | 2 | 0 | 1 | 1 | 0 | 1 | 0 |
| **Stomach ache** | 0 | 0 | 0 | 2 | 1 | 0 | 0 | 0 |
| **Stroke** | 0 | 0 | 0 | 0 | 0 | 0 | 1 | 0 |
| **Thrombo-cytopenia** | 0 | 1 | 0 | 0 | 0 | 1 | 0 | 0 |
| **Uraemia** | 0 | 0 | 0 | 0 | 0 | 0 | 1 | 0 |
| **Unknown** | 0 | 0 | 1 | 2 | 0 | 0 | 1 | 0 |
| **Total** | 11  (15.5%) | 7  (8.9%) | 5  (8.6%) | 10  (6.7%) | 34  (6.6%) | 17  (6.5%) | 15  (6.0%) | 7  (9.6%) |

D2T-RA, difficult-to-treat rheumatoid arthritis; b/tsDMARD, targeted synthetic disease-modifying anti-rheumatic drugs; I TNFi, tumour necrosis factor inhibitor; IL-6Ri,interleukin-6 receptor inhibitor; CTLA4-Ig,cytotoxic T-lymphocyte–associated antigen-4 immunoglobulin; JAKi, Janus kinase inhibitor; LPD, Lymphoproliferative disorder; CHF: congestive heart disease; CK, creatinine kinase; LOC: loss of consciousness.
